# Short-term complication rates of open reduction and plate fixation and intramedullary nailing in the treatment of humeral shaft fractures: a propensity score matched analysis

**DOI:** 10.1007/s00402-024-05491-3

**Published:** 2024-08-09

**Authors:** Sarah Whitaker, Sarah Cole, Conor O’Neill, James Satalich, R. Cole Schmidt, Jennifer Vanderbeck

**Affiliations:** 1 Virginia Commonwealth University School of Medicine, Richmond, VA USA; 2 Department of Orthopaedic Surgery, Virginia Commonwealth University Health System, Richmond, VA USA; 3 Department of Orthopaedic Surgery, Duke University Health System, Durham, NC USA

**Keywords:** Humeral fracture, ORIF, Intramedullary nailing, NSQIP

## Abstract

**Introduction:**

This is a retrospective cohort study designed to compare short-term postoperative complication rates between closed humeral shaft fractures treated by open reduction and internal fixation (ORIF) versus intramedullary nailing (IMN), as well as secondary independent risk factors for adverse outcomes.

**Materials and methods:**

The American College of Surgeons National Surgical Quality Improvement Program (ACS-NSQIP) database was queried using CPT codes to identify patients that underwent an open reduction and plate fixation or intramedullary nailing procedure for a closed humeral shaft fracture from 2010 to 2021. Cohorts were matched using propensity scores to account for demographic differences and rates of complications were compared between the two groups.

**Results:**

From the database, a total of 4,222 patients were identified who met inclusion criteria, with 3,326 and 896 undergoing ORIF and IMN respectively. After propensity score matching, 866 of the nearest-neighbor matches were included in each cohort for a total of 1,732 patients in the final analysis. The rate of any adverse event (AAE) was significantly higher in the ORIF cohort (16.3%) than the IMN cohort (12.1%, *p* = 0.01). The ORIF group had higher rates of postoperative transfusion (*p* = 0.002), return to OR (*p* = 0.005), and surgical site infection (SSI, *p* = 0.03). After multivariate analysis, ASA class 4, increasing age, increasing operative time, and history of bleeding disorder were found to increase the risk of AAE in both ORIF and IMN patients.

**Conclusions:**

While prior studies have claimed higher complication rates in IMN patients, this study found a significantly higher short-term risk of AAE in ORIF patients when compared in matched cohorts. However, individual 30-day complication rates do not differ significantly between procedures, and both have been shown to be safe and effective tools in the management of humeral shaft fractures.

**Supplementary Information:**

The online version contains supplementary material available at 10.1007/s00402-024-05491-3.

## Introduction

Fractures of the humeral diaphysis comprise approximately 30% of humeral fractures and 3% of all fractures [1–3]. While many humeral shaft fractures are managed conservatively, when operative intervention is indicated, often either ORIF or IMN are viable fixation options. While ORIF is the most common method of surgical management for humeral shaft fractures, IMN has risen in popularity and is especially appealing in patients with polytrauma, pathological fractures, or osteoporotic fractures due to being a load-sharing implant with reduced destruction of surrounding soft tissue structures and preserved periosteal blood supply [2, 4, 5]. The decision between the two implants may become more important, as it has been shown that 25% of closed, displaced humeral shaft fractures treated nonoperatively went on to require surgery 12 months later for nonunion and ultimately achieved poorer overall outcome scores compared to individuals who initially underwent surgical fixation [6].

Literature comparing the outcomes of IMN and ORIF is mixed -- some studies have found no difference in operative statistics, functional outcomes, and union time, while others have noted worse outcomes in terms of postoperative complications such as reoperation, shoulder impingement, nonunion, and restricted range of motion with IMN [2, 7–12]. Multiple studies have cited an increased risk of postoperative radial nerve palsy following ORIF [2, 8, 13] while others have found no significant difference [3, 7, 9]. Other studies have noted statistically significant differences in these variables between IMN and ORIF with some finding worse outcomes with IMN [2, 3, 11, 12]. Other postoperative complications such as infection or blood loss also have contradictory findings, with several studies reporting ORIF to be a safer procedure overall in terms of increased risk of any postoperative complication, reoperation, shoulder impingement, restricted range of motion, and iatrogenic fractures [3, 8, 14, 15], and others finding IMN to have faster rates of union, lower infection rates, shorter operation times, less blood loss, and lower risk of severe complications [7, 9, 13].

Two prior studies have compared postoperative complications and patient demographics between ORIF and IMN using the NSQIP dataset [16, 17]. Putnam et al. concluded that there were no significant differences in terms of major or minor complications, length of stay, or readmission, but did find higher rates of mortality in the IMN cohort [16]. However, this publication only considered NSQIP cases performed between 2005 and 2016 and performed propensity score matching that did not take into account body mass index (BMI), race, bleeding disorders, diabetes, and smoking status. They did include peripheral nerve injury as a postoperative complication in their data, however, NSQIP guidelines that state this is an unreliable outcome to assess from their dataset after 2010 [16, 18]. The data from Burgmeier et al. agreed that IMN demonstrated a significantly higher postoperative mortality rate, and the authors theorized this finding could be due to the difference in age between cohorts, as they did not utilize a propensity score match in their analysis [17]. Similar to Putnam et al., they only extracted data from the NSQIP datasets from 2007 to 2015 whereas an additional 1,083 IMN and 2,989 ORIF have been recorded in the datasets from 2016 to 2021.

The primary objective of this retrospective comparative study is to utilize a large dataset with matched cohorts to compare 30-day postoperative complication rates between closed humeral shaft fractures treated with ORIF to those treated with IMN. Lastly, we will aim to identify independent risk factors for adverse events in surgically treated humeral shaft fractures.

## Methods

To assess short-term outcomes between ORIF and IMN procedures for humeral shaft fractures, this study retrospectively reviewed data collected as a part of the ACS NSQIP database. Access to this information was provided by the ACS. This database gathers surgical data from a multitude of participating hospitals through a trained clinical nurse reviewer who collects prospective information on patient demographics, surgical procedure(s) and outcomes, concomitant conditions and risk factors, laboratory results, and 30-day complication rates [19]. Among participating institutions, the collection methods are standardized and require an interrater reliability rate of less than 5% at each site [20]. Prior studies have demonstrated less than 2% interrater disagreement rates, and sites are subject to routine audits for quality assurance [21, 22]. Overall, the NSQIP database has proven to be a reliable source of information in orthopedic and fracture fixation research [16, 17, 20, 21, 23, 24].

In order to select patients for this study, the database was filtered using Current Procedure Terminology (CPT) codes to only select individuals who had undergone either an ORIF (24515) or IMN (24516) between the years of 2010 and 2021. Individuals were excluded on the basis of International Classification of Diseases Ninth and Tenth Revision (ICD-9, ICD-10) if they had one of the above surgeries for open humeral shaft fracture, stress fracture, pathological fracture, neoplastic disease, or metastatic disease or if they did not have an ICD diagnosis code for a humerus fracture. An additional six patients were found to have CPT codes for both ORIF and IMN and were thus removed from the study.

Once ORIF and IMN cohorts had been formed, a 1:1 propensity match was performed to account for bias in treatment assignment and imitate randomization. This matched subjects in each cohort on the basis of age, sex at birth, body mass index (BMI), American Society of Anesthesiologists (ASA) physical status classification score, diabetes mellitus, bleeding disorders, hypertension requiring medication, steroid use, functional status, congestive heart failure (CHF), smoking status, and chronic obstructive pulmonary disease (COPD). Each matched patient was then evaluated for length of stay, 30-day complications, and readmission rate. Complications included for evaluation consisted of wound dehiscence, superficial and deep surgical site infections (SSI), intubation complications including failure to wean or re-intubation postoperatively, deep vein thrombosis (DVT), cardiac arrest, urinary tract infection, myocardial infarction (MI), cerebrovascular accident (CVA), pulmonary embolism (PE), postoperative transfusions, renal complications including insufficiency or failure, pneumonia, sepsis, and death. In addition to these variables, length of stay (LOS) from procedure to discharge above the mean value were analyzed.

To evaluate the significance of the results, statistical analyses were performed using RStudio software version 2023.06.1 + 524 (R Foundation for Statistical Computing, Vienna, Austria) with a variety of strategies employed including multivariate and bivariate analysis and propensity score matching. Patient demographics, risk factors, and complications in both the unmatched and matched cohorts were analyzed with two-tailed T-tests for continuous variables or with chi-square tests for categorical variables. Depending on the outcomes of univariate testing, complication variables in the matched cohorts with a p value less than 0.05 were further compared using multivariable logistic regression analysis. Variables with a p-value less than 0.05 were considered to be statistically significant.

## Results

### Demographics

A total of 4,222 patients who met inclusion/exclusion criteria were identified, of which 3,326 (78.8%) underwent ORIF and 896 (21.2%) underwent IMN (Table 1). Prior to matching, there were statistically significant differences in age, sex, BMI, operative time, length of hospital stay, outpatient status, ASA classification, estimated probabilities of morbidity and mortality, functional status, requirement of pre-operative blood transfusion, and history of dialysis, bleeding disorders, diabetes, COPD, and preoperative steroid use. Complete demographic information is outlined in Table 1.

**Table 1:** Demographic and comorbidity characteristics for patients undergoing open reduction and internal fixation (ORIF) and intramedullary nailing (IMN) for treatment of closed humeral shaft fractures

		ORIF Unmatched (%)	IMN Unmatched (%)	*p*-value	ORIF Matched (%)	IMN Matched (%)	*p*-value
Patients, *N* (%)		3326 (78.8)	896 (21.2)		866 (50)	866 (50)	
Age (years, mean ± SD)		55.1 ± 20.7	64.9 ± 16.2	**< 0.001**	64.5 ± 16.4	64.1 ± 15.8	0.609
BMI (kg/m^2^, mean ± SD)		30.3 ± 8.11	29.6 ± 7.91	**0.0334**	30.4 ± 7.75	29.8 ± 7.95	0.0883
Male sex (%)		1251 (37.6)	286 (31.9)	**0.0019**	265 (30.6)	281 (32.4)	0.438
Operative Time (mins)		131.8 ± 62.8	100.7 ± 54.9	**< 0.001**	129.5 ± 61.9	101.5 ± 55.1	**< 0.001**
Length of Stay		2.31 ± 3.85	3.16 ± 5.39	**< 0.001**	2.94 ± 4.11	3.16 ± 5.46	0.343
Outpatient status		1590 (47.8)	382 (42.6)	**0.00661**	365 (42.1)	377 (43.5)	0.593
ASA Class		2.35 ± 0.76	2.67 ± 0.68	**< 0.001**	2.67 ± 0.67	2.66 ± 0.69	0.750
1 (no disturbance)		450 (13.5)	31 (3.46)	**-**	29 (3.35)	31 (3.58)	**-**
2 (mild disturbance)		1409 (42.4)	312 (34.8)	**-**	298 (34.4)	309 (35.7)	**-**
3 (severe disturbance)		1328 (39.9)	474 (52.9)	**-**	470 (54.3)	451 (52.1)	**-**
4 (life-threatening disturbance)		139 (4.18)	79 (8.82)	**-**	69 (7.97)	75 (8.66)	**-**
5 (moribund)		0	0		0	0	**-**
Race							
White		2338 (70.3)	712 (79.5)	**-**	689 (79.6)	686 (79.2)	**-**
Black		258 (7.76)	48 (5.36)	**-**	56 (6.47)	46 (5.31)	**-**
Asian		85 (2.56)	17 (1.90)	**-**	12 (1.39)	17 (1.96)	**-**
Other		33 (0.99)	9 (10.0)	**-**	7 (0.81)	9 (1.04)	**-**
Unknown		612 (18.4)	110 (12.3)	**-**	102 (11.8)	108 (12.5)	**-**
Dependent functional status (partial or total)		174 (5.23)	88 (9.82)	**< 0.001**	74 (8.55)	75 (8.66)	1
Current smoker		656 (19.7)	179 (20.0)	0.903	175 (20.2)	179 (20.7)	0.858
Comorbidities *N* (%)							
Congestive heart failure		37 (1.11)	14 (1.56)	0.356	14 (1.62)	13 (1.50)	1
Dialysis		16 (0.48)	12 (1.34)	**0.00996**	7 (0.81)	12 (1.39)	0.356
Steroid use		91 (2.74)	42 (4.69)	**0.00423**	38 (4.39)	42 (4.85)	0.731
Bleeding disorder		134 (4.03)	52 (5.80)	**0.0274**	51 (5.89)	49 (5.66)	0.918
Ascites		3 (0.090)	2 (0.22)	0.631	2 (0.23)	2 (0.23)	1
Pre-operative transfusion		49 (1.47)	24 (2.68)	**0.0208**	20 (2.31)	22 (2.54)	0.876
Diabetes		615 (18.5)	201 (22.4)	**0.00919**	208 (24.0)	201 (23.2)	0.734
IDDM		253 (7.61)	93 (10.4)	**-**	103 (11.9)	93 (10.7)	**-**
NIDDM		362 (10.9)	108 (12.1)	**-**	105 (12.1)	108 (12.5)	**-**
COPD		134 (4.03)	65 (7.25)	**< 0.001**	61 (7.04)	62 (7.16)	1

BMI: body mass index; COPD: chronic obstructive pulmonary disease; Dialysis: acute or chronic renal failure requiring dialysis within 2 weeks of indexed procedure; IDDM: insulin-dependent diabetes mellitus

After matching, 866 patients were included in analysis for both the IMN cohort and the ORIF cohort, for a total of 1,732 patients in the final analysis (Table 1). The average age across both cohorts was 64.3 years, average BMI was 30.1, and 31.5% of patients were male. No baseline demographic characteristics varied significantly between groups.

### Outcomes

Detailed outcome information is presented in Table 2. After matching, the ORIF cohort had a significantly higher overall rate of adverse events than the IMN cohort (16.3% vs. 12.1%, *p* = 0.01) (Table 2). The ORIF cohort had a higher rate of postoperative blood loss requiring transfusions (11.9% vs. 6.6%, *p* = 0.002), surgical site infections (SSI) (1.9% vs. 0.7%, *p* = 0.03), and return to OR (3.8% vs. 2.2%, *p* = 0.04). There were no significant differences in the other adverse events recorded. Additionally, the average operative time in the IMN cohort was significantly lower compared to the ORIF cohort (101.5 ± 55.1 vs. 129.5 ± 61.9, *p* < 0.001). There were no differences in length of stay or outpatient status between groups.

**Table 2:** Incidence of adverse events for patients undergoing ORIF vs. IMN

	ORIF Unmatched		IMN Unmatched			ORIF Matched		IMN Matched			Overall Matched	
	No.	Rate (%)	No.	Rate	*p*-value	No.	Rate	No.	Rate	*p*-value	No.	Rate (%)
Any adverse event	355	10.7	112	12.5	0.137	141	16.3	105	12.1	**0.0132**	246	14.2
Death	17	0.51	17	1.90	**0.0034**	5	0.58	12	1.39	0.0881	17	0.98
Wound dehiscence	5	0.15	1	0.11	0.766	3	0.35	1	0.12	0.317	4	0.23
Sepsis	20	0.60	7	0.78	0.578	11	1.27	6	0.69	0.223	17	0.98
Pulmonary embolism	10	0.30	3	0.33	0.874	2	0.23	2	0.23	1	4	0.23
Acute renal failure	7	0.21	1	0.11	0.471	4	0.46	1	0.12	0.179	5	0.29
Myocardial infarction	7	0.21	2	0.22	0.943	5	0.58	2	0.23	0.256	7	0.40
Cardiac arrest	3	0.090	2	0.22	0.424	2	0.23	2	0.23	1	4	0.23
Stroke	2	0.060	0	0	0.157	0	0	0	0	1	0	0
Transfusion	207	6.22	53	5.92	0.730	88	11.9	52	6.57	**0.0015**	140	8.08
DVT	11	0.33	5	0.56	0.397	4	0.46	4	0.46	1	8	0.46
UTI	24	0.72	12	1.34	0.133	9	1.04	12	1.39	0.510	21	1.21
Pneumonia	19	0.57	8	0.89	0.345	9	1.04	8	0.92	0.808	17	0.98
Intubation issues	12	0.36	4	0.45	0.728	6	0.69	4	0.46	0.526	10	0.58
SSI	49	1.47	6	0.67	**0.0194**	16	1.85	6	0.69	**0.0319**	22	1.27
Return to the OR	75	2.25	21	2.34	0.876	33	3.81	19	2.19	**0.0487**	52	3.00
Extended LOS	259	7.79	117	13.1	**< 0.001**	107	12.4	115	13.3	0.566	222	12.8

Any adverse event (AAE): superficial and deep surgical site infection, organ space infection, wound dehiscence, renal failure, intubation (fail to wean or reintubation), post-operative transfusion, pneumonia, DVT, PE, UTI, stroke, cardiac arrest, MI, return to the OR, death; DVT: deep vein thrombosis; UTI: urinary tract infection; SSI: surgical site infection; LOS: Length of stay (extended: greater than 1 standard deviation above the mean); OR: operating room; Intubation issues: re-intubation or failure to wean from intubation

When accounting for all other variables, ASA 4 classification (OR = 1.197; CI = 1.073–1.335) and history of bleeding disorder (OR = 1.149; CI = 1.072–1.231) increased the odds of any adverse event postoperatively (Table 3). Additionally, increasing age (in 1-year intervals) (OR = 1.002; CI = 1.001–1.004) and increasing operative time (in 1-minute intervals) (OR = 1.001; CI = 1.0006–1.0011) modestly increased the risk of developing any adverse event postoperatively. These findings correspond with a 2.02% increase in adverse event rate with every additional 10 years of age and a 1.5% increase in adverse event rate with every additional 15 min of operative time.

**Table 3:** Odds of developing any adverse event during surgery as related to patient demographics, comorbidities, and procedure

Multivariable Analysis**				
		OR Coef.	95% CI	*P*-value
Overall				
Age (1-year intervals)		1.002	1.001–1.004	**< 0.001**
Operative Time (1 min intervals)		1.001	1.0006–1.0011	**< 0.001**
Sex				
Female		Ref	-	-
Male		0.994	0.959–1.029	0.716
ASA class				
1		Ref	-	-
2		0.956	0.873–1.048	0.336
3		1.043	0.949–1.146	0.384
4		1.197	1.073–1.335	**0.0013**
Diabetes mellitus				
IDDM		Ref	-	-
NIDDM		1.023	0.959–1.092	0.486
None		0.999	0.948–1.052	0.971
COPD				
No		Ref	-	-
Yes		0.995	0.935–1.060	0.883
Bleeding Disorder				
No		Ref	-	-
Yes		1.149	1.072–1.231	**< 0.001**
Steroid Use				
No		Ref	-	-
Yes		0.963	0.893–1.039	0.333

** Variables are adjusted for all baseline characteristics; Reference procedure: ORIF

OR: Odds Ratio; Coef: coefficient; 95% CI: 95% Confidence Interval; Ref: reference; ASA: American Society of Anesthesiology; NIDDM: Non-Insulin Dependent Diabetes Mellitus; IDDM: Insulin-Dependent Diabetes Mellitus

## Discussion

Current literature on short-term complications rates following ORIF versus IMN is largely limited to systematic reviews and meta-analyses of small cohort studies, and the few large, matched cohort studies preceding this one utilized older data without strong propensity matching. Our study showed a statistically significant increased rate of overall adverse events in patients who underwent ORIF as opposed to IMN, largely attributable to significantly higher rates of blood transfusion. On univariate analysis, ORIF was found to have higher rates of SSI, postoperative blood loss requiring transfusion, and return to the operating room.

Overall rates of all adverse events were 16.3% for ORIF and 12.1% for IMN. This is in line with previously quoted complication rates of 2.83-20.0% for ORIF [16, 17, 25–28]. These results support several recent studies who found that intramedullary nailing may be preferable over ORIF for management of humeral shaft fractures due to shorter time to union and lower rates of postoperative infection and iatrogenic radial nerve palsy [2, 9, 13, 29]. When analyzing specific complications, rates of postoperative blood loss requiring transfusion after ORIF were nearly twice as high as IMN. This is supported by recent findings by Beeres et al. who found decreased postoperative blood loss in patients who received IMN versus open plate fixation for humeral shaft fractures [13]. Our findings contradict, however, prior studies which report transfusion rates of 4.8-8.0% for ORIF [16, 17, 25, 27, 28] and 10.97-12.0% for IMN [16, 17]. This is likely due to a paucity of current research on short-term complication rates after IMN for proximal humerus fractures, with only Putnam et al. and Burgmeier et al. reporting transfusion rates following IMN. Both of these studies utilize data from an earlier time frame − 2007–2015 for Burgmeier et al. and 2005–2016 for Putnam et al. [16, 17]. Given that IMN has risen in popularity in recent years, the results of these studies are likely not representative of current complication rates following IMN. In addition, our study found that the rate of surgical site infection was significantly higher in the ORIF cohort. This is similar to findings by recent studies which report postoperative infection rates to be 0.5–6.8% for ORIF and 0.2–1.9% for IMN [2, 13, 29, 30]. Finally, more patients receiving ORIF returned to the operating room within 30 days when compared to patients receiving IMN. This supports recent studies with quoted reintervention rates of 1.7–12.6% for ORIF [13, 17, 25, 27, 28] and 1.2–9.7% for IMN [13, 17, 30].

Our study found no significant difference in mortality rates between the matched ORIF and IMN cohorts. This contradicts assertions by Burgmeier et al. and Putnam et al., who also utilized the ACS NSQIP database to compare ORIF and IMN outcomes. Both studies found significantly increased mortality rates in the IMN group compared to the ORIF group [16, 17]. However, both Burgmeier’s and Putnam’s studies used data from early time periods (2007–2015 and 2005–2016, respectively) and either did not utilize propensity score matching or utilized an algorithm that excluded key demographics, including BMI, smoking status, and more [16, 17]. Therefore, we believe these studies may be underpowered in their comparison of current mortality rates of ORIF and IMN. It is worth noting that prior to matching, the IMN cohort had significantly higher rates of several comorbidities, indicating that surgeons were likely opting for IMN over ORIF in high-risk patients, which may partly explain Putnam’s and Burgmeier’s findings.

Our study found increasing age and operative time to be independent risk factors for any adverse event (Table 3). While these results are statistically significant, their clinical significance appears to be modest. An ASA 4 classification and history of bleeding disorder were also associated with worse outcomes in the ORIF cohort (Table 3). This is consistent with studies analyzing predictive variables associated with major complications following shoulder surgery, which found increasing ASA classification to be an independent risk factor. [31, 32] These are important findings to consider on a case-by-case basis when determining a patient’s best option for treatment.

This study has several limitations. First, outcome variables are limited in the NSQIP database. While recent studies have focused on nonunion rates and time to union as primary outcomes when comparing IMN to open plate fixation, the NSQIP database does not include these variables as outcome data is limited to 30 days postoperatively. Therefore, no conclusions can be made with this data on nonunion rates or time to union. Meta-analyses by Amer et al., Beeres et al., and Wen et al. found no significant difference in nonunion rates between groups [2, 9, 13], but additional research using large, matched cohorts is needed. Second, our study did not investigate radial nerve palsy as an outcome due to lack of reliable data on peripheral nerve injury in the NSQIP database. The rate of iatrogenic radial nerve palsy following surgical treatment of humeral fractures is estimated to be between 3 and 19% based on recent studies [4, 15, 33, 34]. However, it is currently unknown whether the risk of iatrogenic radial nerve injury varies significantly between patients receiving IMN versus plate fixation [2, 3, 9, 13]. A third limitation of this study is the limited time period of postoperative follow-up. As previously stated, the NSQIP database only includes data for the 30-day postoperative period. However, short-term complication rates can be useful in predicting long-term outcomes. Additionally, patients with humeral shaft fractures requiring fixation are often multiply injured due to trauma [4]. Therefore, it is important to investigate not only long-term efficacy of fixation methods for such injuries, but also the incidence of severe, life-threatening short-term complications. Such investigation is critical for surgeons choosing a surgical approach and providing patients with accurate rates of severe complications. Therefore, despite the limitations of the NSQIP database in analyzing long-term outcomes and orthopedic-specific complications, the data it provides can help surgeons in their clinical decision making and during the informed consent process. Finally, our study does not explore newer operative options for humeral shaft fractures, namely minimally invasive plate osteosynthesis (MIPO). Despite these limitations, our strict inclusion criteria, large patient number, and utilization of matched cohorts may improve research validity and provide more specific information for physicians considering treatment options for humeral shaft fractures.

## Conclusion

This study found that patients undergoing ORIF for closed humeral shaft fractures have higher rates of overall short-term complications and, in particular, higher rates of blood transfusion, surgical site infection, and return to the operating room. Discussion surrounding operative treatment of humeral shaft fractures has recently favored IMN as the safer route, but most recent studies have not utilized propensity score matching. Therefore, this conclusion may not hold true when accounting for baseline demographic differences. Our investigation found that the overall adverse event risk was higher in patients receiving ORIF compared to IMN, though most outcomes do not vary significantly when analyzed individually. This study presents important and relevant data for surgeons to consider in clinical decision making for treatment of closed humeral shaft fractures.

## Electronic supplementary material

Below is the link to the electronic supplementary material.

Supplementary Material 1
