# Supplementary material for: Short-term complication rates of open reduction and plate fixation and intramedullary nailing in the treatment of humeral shaft fractures: a propensity score matched analysis
Source: Arch Orthop Trauma Surg. 2024 Aug 9;144(8):3361–8. doi: 10.1007/s00402-024-05491-3 (PMC11417072; doi:10.1007/s00402-024-05491-3)
Supplement: Supplementary file 1 — Supplementary Material 1 [file 402_2024_5491_MOESM1_ESM.docx]

**Title:** Short-Term Complication Rates of Open Reduction and Plate Fixation and Intramedullary Nailing in the Treatment of Humeral Shaft Fractures: A Propensity Score Matched Analysis

**Authors:** Sarah Whitaker^a^ BA, Sarah Cole^a^ BA, Conor O’Neill^c^ MD, James Satalich^b^ MD, R. Cole Schmidt^b^ MD, Jennifer Vanderbeck^b^ MD

^a^Virginia Commonwealth University School of Medicine, Richmond, VA, USA

^b^Department of Orthopaedic Surgery, Virginia Commonwealth University Health System, Richmond, VA, USA

^c^Department of Orthopaedic Surgery, Duke University Health System, Durham, NC, USA

**Corresponding author:** Sarah Whitaker

(804) 971-2046

whitakerst@vcu.edu

**Disclosures**: No potential conflict of interest relevant to this article was reported.

**Funding:** The authors have no relevant financial or non-financial interests to disclose.
